# Integration of lead-free ferroelectric on HfO_2_/Si (100) for high performance non-volatile memory applications

**DOI:** 10.1038/srep08494

**Published:** 2015-02-16

**Authors:** Souvik Kundu, Deepam Maurya, Michael Clavel, Yuan Zhou, Nripendra N. Halder, Mantu K. Hudait, Pallab Banerji, Shashank Priya

**Affiliations:** 1 Center for Energy Harvesting Materials and Systems (CEHMS), Department of Mechanical Engineering, Virginia Tech, Blacksburg, Virginia 24061, USA; 2 Advanced Devices & Sustainable Energy Laboratory (ADSEL), Bradley Department of Electrical and Computer Engineering, Virginia Tech, Blacksburg, Virginia 24061, USA; 3 Advanced Technology Development Centre, Indian Institute of Technology Kharagpur, Kharagpur 721302, India; 4 Materials Science Centre, Indian Institute of Technology Kharagpur, Kharagpur 721302, India

## Abstract

We introduce a novel lead-free ferroelectric thin film (1-x)BaTiO_3_-xBa(Cu_1/3_Nb_2/3_)O_3_ (x = 0.025) (BT-BCN) integrated on to HfO_2_ buffered Si for non-volatile memory (NVM) applications. Piezoelectric force microscopy (PFM), x-ray diffraction, and high resolution transmission electron microscopy were employed to establish the ferroelectricity in BT-BCN thin films. PFM study reveals that the domains reversal occurs with 180° phase change by applying external voltage, demonstrating its effectiveness for NVM device applications. X-ray photoelectron microscopy was used to investigate the band alignments between atomic layer deposited HfO_2_ and pulsed laser deposited BT-BCN films. Programming and erasing operations were explained on the basis of band-alignments. The structure offers large memory window, low leakage current, and high and low capacitance values that were easily distinguishable even after ~10^6^ s, indicating strong charge storage potential. This study explains a new approach towards the realization of ferroelectric based memory devices integrated on Si platform and also opens up a new possibility to embed the system within current complementary metal-oxide-semiconductor processing technology.

Ferroelectric materials based non-volatile memory (NVM) devices are being investigated for ferroelectric random access memory (FeRAM) applications due to their unlimited write cycles, high speed, longer life times, high integration density and low power consumption compared with the flash and conventional dynamic random access memory (DRAM)^1^,^2^,^3^,^4^. Ferroelectric materials are spontaneously polarized and can store information on the basis of their remnant polarization, i.e., polarization remains in the material even in the absence of electric field. Therefore, it has a great potential for use in NVM devices. Monolithic integration of ferroelectric materials on silicon (Si) opens up a new pathway towards developing advanced Si-based NVM, sensors, optoelectronic, and logic devices^2^. However, integration of such materials onto Si is a very challenging task due to the possibility of cross interdiffusion at high temperature. Ferroelectric integration on closely lattice mismatched NdGaO_3_ or SrTiO_3_ substrates is an alternative approach towards fabrication of electronic devices, however, these substrates cannot be embedded into complementary metal-oxide-semiconductor (CMOS) systems for further device processing since current CMOS processing technologies are based on low-cost, large area Si^5^. Many researchers have attempted to implement the ferroelectric materials onto Si, but direct integration results in cross-diffusion that creates defect states and degrades the electrical transport properties^3^,^4^,^6^,^7^,^8^,^9^,^10^,^11^,^12^,^13^. Moreover, an interfacial reaction between the ferroelectric layer and the Si can provide poor charge retention property which ultimately hinders the device performance. To alleviate these problems, a high-k barrier layer was introduced by several researchers between the ferroelectric and the Si substrate^3^,^4^,^6^,^7^,^8^,^9^,^10^,^11^,^12^,^13^. The limitations of this approach are the presence of depolarization field across the ferroelectric layer and the large voltage drop across the oxide layer, which results in a poor device performance and also increases the device operation voltage^13^. The depolarization voltage implies polarization induced by an external field opposing the applied field and it could seriously affect the retention property of the NVM devices. In addition, the presence of a depolarization field can also affect the orientation of ferroelectric domains which may suppress the ferroelectric switching^2^. It is known that the performance of such NVM devices strongly depends on the switching of the ferroelectric polarization, since it can create high memory densities, as well as improve the retention properties^2^,^14^. The switching of ferroelectric domains, through nucleation and growth, occurs in presence of an external bias. In ferroelectric material, mechanical strain and depolarization field have been found to have a large impact on the formation of polarization domains. When one wants to integrate ferroelectric thin films with Si, the domain formation is controlled by the lattice mismatch between the thin films and the substrate and interfacial defects could also arise during processing steps^15^.

In ferroelectric materials, domain structure affects its piezoelectricity, hysteresis and switching behavior^14^. It is also understood that in order to investigate the dielectric properties, 180° domain inversion is particularly important^2^,^16^,^17^. Thus, investigation of domain formation and demonstration of polarization inversion offers a promising approach towards design of a futuristic ferroelectric based NVM devices. It is noteworthy to mention that the performance of such memory devices can be emphasized by investigating the three important parameters; memory window (MW), leakage current, and charge retention time. Although, many researchers have investigated the ferroelectric based NVM devices onto Si platform, but not much work has been performed in elucidating the correlation between the domain nucleation and growth in ferroelectric films for NVM device and switching hysteresis^3^,^4^,^6^,^7^,^8^,^9^,^10^,^11^,^12^,^13^. It is important to quantify the behavior of domains and perform switching measurements in order to explain the macroscale electrical properties^2^,^17^,^18^. The electrical properties reported in prior studies are not sufficient to meet the criterion for the FeRAM applications (see Supplementary Table S1 online). The lack of information on domain kinetics combined with the limited understanding of the device efficiencies to perform write-read-erase operations demands a systematic study that can address these facets^2^. Recently Dubourdieu et al. demonstrated switching of ferroelectric polarization on Si and suggested that their system can be suitable for fabricating NVM devices^2^. However, to design a high performance ferroelectric memory device one has to improve the electrical parameters as described above.

BaTiO_3_ (BTO) has been considered to be an attractive candidate for the fabrication of NVM devices due to its strong piezoelectric effect and ferroelectric switching^19^. For bulk BTO, the tetragonal phase is stable at room temperature whereas the thin films could adopt the cubic phase depending upon the magnitude of interfacial stress^19^. Recently, Yan et al. improved the electrical properties of BTO by doping with Co and reported a high performance resistive memory device^20^. There is a significant scope to improve both the ferroelectric and electrical properties of BTO by carefully tuning the isovalent substitution of Ti with proper doping materials. In our earlier work, we have demonstrated that blending of complex dilute solutions of Ba(Cu_1/3_Nb_2/3_)O_3_(BCN) into BaTiO_3_ given by composition (1-x)BaTiO_3_-xBa(Cu_1/3_Nb_2/3_)O_3_ (where, x = 0.025, BT-BCN) resulted in low loss and high piezoelectric response^21^,^22^,^23^. The bulk BT-BCN exhibits low coercive field, high remnant polarization, and excellent switching and hysteresis behavior. *In the present study*, for the first time we investigate the ferroelectric property of BT-BCN thin films heterogeneously integrated with Si using HfO_2_ buffer layer. Piezoelectric force microscopy (PFM) was employed to understand the domain switching and high-resolution transmission electron microscopy (HRTEM) was used to analyze the BT-BCN domains and interface with atomic layer deposited HfO_2_. The band-alignments between the BT-BCN and the HfO_2_ layer is indispensable in order to describe the operation principle of Al/BT-BCN/HfO_2_/p-Si NVM devices. This band alignment was quantified using x-ray photoelectron spectroscopy (XPS) measurement. The electronic band-diagrams of these memory devices during programming and erasing operations are proposed and a systematic investigation is conducted on the electrical performance of these devices.

## Results

### Investigation of ferroelectricity in BT-BCN thin films

**Figure 1:**
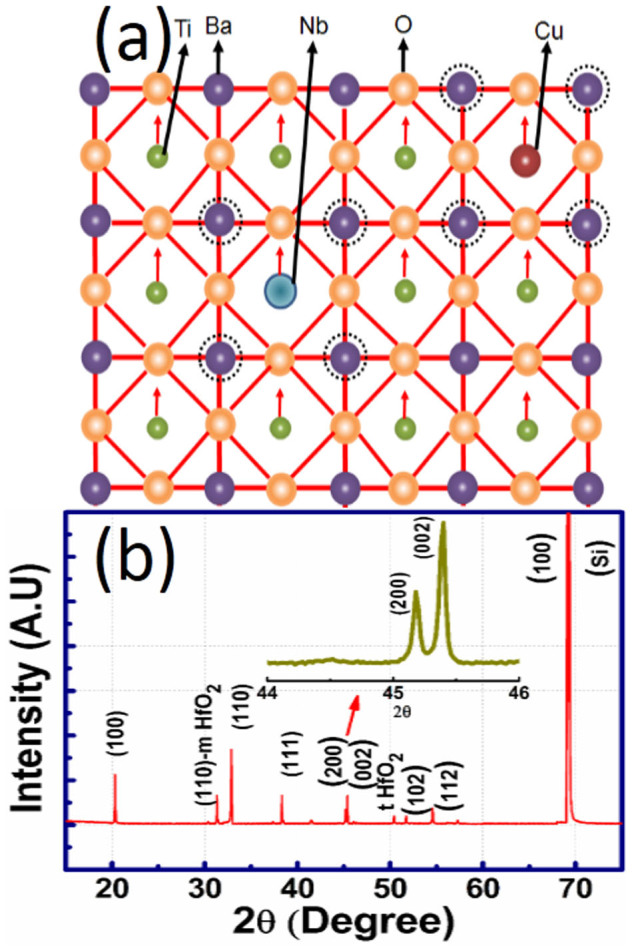
Structural analysis of BT-BCN. (a) The substitution of Nb and Cu on the Ti site in BaTiO_3_ increases piezoelectric response. Ba site off centering causes formation of local polar region with lower symmetry due to increased interaction between the Ba-Nb and Ba-Cu compared with Ba-Ti, and octahedral symmetry of the copper complexes suggests that copper occupies Ti^4+^ site with divalent Cu^2+^ oxidation state. The random distribution of these structural distortions can lead to improve polarization in BT-BCN; and (b) The x-ray diffraction of BT-BCN on HfO_2_/Si in room temperature. (inset) The Bragg reflection in XRD confirms the tetragonal structure.

It was found that in BT-BCN bulk system, the ferroelectric polarization or piezoelectric response increases with the value of x reaching maxima at x = 0.025, and then starts to decrease^21^. Therefore, we have incisively synthesized BT-BCN with x = 0.025 for thin film deposition. In the BT-BCN material structure, the substitution of Nb (ionic radii = 0.69 Å) and Cu (ionic radii = 0.73 Å) on the Ti-site (ionic radii = 0.60 Å) in BaTiO_3_ can induce local polar region with lower symmetry (as shown in Fig. 1(a)). The substitution of these larger ions will also introduce the higher repulsive interaction between Ba-Nb and Ba-Cu compared with Ba-Ti (the values of ionic radii are in six-fold coordination). The octahedral symmetry of the copper complexes suggests that copper occupies Ti^4+^ site with divalent Cu^2+^ oxidation state, i.e., these substitutions are expected to promote A-site off centering, which helps to form the polar nano regions and also facilitates in lowering of local symmetry. The random distribution of these structural distortions can alleviate the polarization rotation and support averaging in anisotropy energy^21^. The XRD pattern of BT-BCN/HfO_2_/Si structure is shown in Fig. 1(b) depicting perovskite structure. The inset shows the presence of (200) and (002) peaks due to BCN modification which confirms the formation of its tetragonal structure^21^. XRD pattern also shows the (110) peak of atomic layer deposited (ALD) HfO_2_ and the (100) peak of Si. The HfO_2_ films grown at 250°C by ALD was amorphous. However, the BT-BCN was deposited onto HfO_2_/Si at 750°C in an O_2_ ambient immediately after deposition of HfO_2_ films. Therefore, the HfO_2_ films also experienced annealing during BT-BCN growth due to the high temperature ambient and O_2_ rich environment. At that high temperature, HfO_2_ becomes crystalline and the XRD spectrum of HfO_2_ reveals the coexistence of both monoclinic and tetragonal phases. No XRD peaks would be seen if the HfO_2_ film was amorphous, which was the case for as grown films sputter-deposited at room temperature^24^. Our results can also be compared with the literature, where HfO_2_ was deposited onto Si and then annealed at different temperatures. Both the monoclinic and tetragonal phases are also present in the HfO_2_/Si XRD spectra when the HfO_2_ was annealed between 700°C and 800°C^24^.

**Figure 2:**
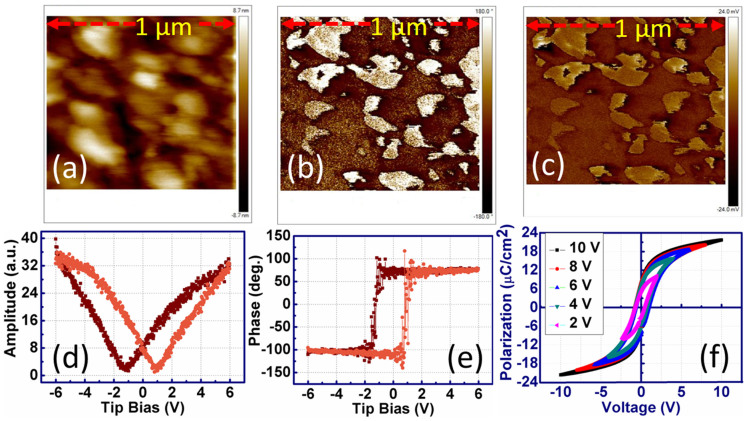
Local hysteresis behavior of BT-BCN thin films (thickness: 300 nm) on HfO_2_/Si. (a) surface topography; (b) PFM amplitude; (c) PFM phase images. Local hysteresis loops measured by PFM (d) amplitude component; (e) polarization-voltage hysteresis loops from microscopic measurements; and (f) polarization-voltage hysteresis loops using top electrode from electrical measurements.

To establish the evidence of ferroelectricity, one has to probe the local domain configurations and demonstrate switching of the polarization^2^,^17^,^18^. The domain structure and its modification with applied electric field will provide direct information on the static and dynamic properties of the ferroelectric^25^. In the presence of an electric field, the formation of ferroelectric domains or phases will influence the memory window or retention property in NVM devices as this parameter is related to the polarization charge^2^,^14^. Figure 2(a) shows the PFM topography obtained from the surface of BT-BCN film. Figures 2(b) and 2(c) show the PFM phase and amplitude micrographs, respectively. These results exhibit 180° domain wall movement between the *c+* (positive polarizations, where surface charge is positive) and *c−* (negative polarizations, where surface charge is negative) domains. In these micrographs, the dark region is polarized downward, whereas, the yellow region is polarized upward, confirming the 180° phase difference between two domain configurations^16^. The out-of-plane piezoresponse was determined as a function of applied voltage and the PFM hysteresis loop both in amplitude and phase are shown in Fig. 2(d) and 2(e), respectively. Figure 2(d) exhibits butterfly shape, whereas Fig. 2(e) reveals a sharp 180° inversion at the coercive voltages with clear hysteresis. All of these results confirm the ferroelectric nature of BT-BCN thin film. From the minima of the amplitude loop, the local coercive voltages are found to be −1.70 and +1 V. Figure 2(f) shows the corresponding electrical P-V hysteresis loop where the remnant polarization and coercive voltage increases with increasing applied voltage from 2 to 10 V. The saturated polarization was found to be 23.5 μC/cm^2^, whereas, the coercive voltage was 1.3 V for 6 V of operation. A slight change in coercive voltage obtained in electrical polarization-voltage (P-V) measurement as compared with PFM P-V measurement is believed to be due to the difference in the electrical boundary conditions at the local top electrodes (Al for electrical P-E and Pt coated tip for PFM P-V)^26^.

**Figure 3:**
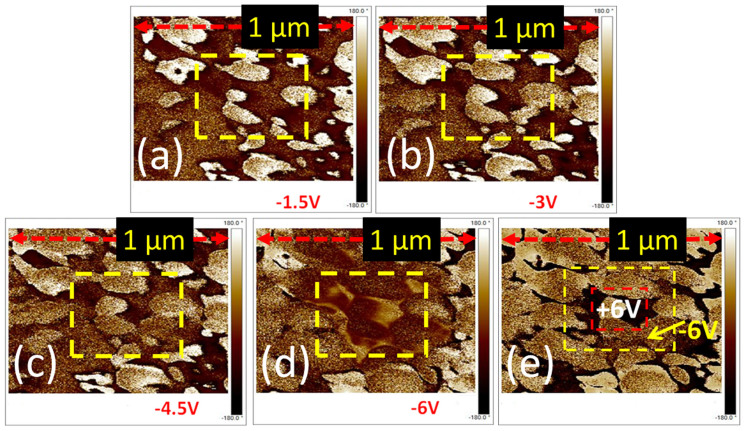
Ferroelectric domain switching observation. Domain switching under negative bias with (a) −1.5, (b) −3, (c) −4.5, and (d) −6 V over 500 nm region. With increasing negative poling voltage from −1.5 V, the dark regions started to switch and at −6 V completely switched into yellow regions inside the square pattern marked in the images, i.e., 180° phase shift has been observed; and (e) Domains switching under positive bias with 6 V over 250 nm region. The yellow region again started to change in dark region when the opposite bias was applied, indicates a clear 180° domains inversion of the phase. Thus, memory rewritable mechanism can be realized in this system through polarization inversion.

In ferroelectric materials, inhomogeneous nucleation and anisotropic growth plays an important role in polarization switching^27^. To explore the reversible polarization switching phenomenon, square polarization pattern (500 × 500 nm^2^) has been generated by scanning the surface of BT-BCN films with an applied dc bias of −1.5 (Fig. 3(a)), −3 (Fig. 3(b)), −4.5 (Fig. 3(c)), and −6 V (Fig. 3(d)), respectively using a Pt coated scanning tip (SCM-PIT, Bruker). Interestingly, with negative poling (−6 V), the dark regions switched into yellow regions inside the square pattern marked in the images, indicating the polarization switching after the application of external electric field. To demonstrate the impact of positive poling, a positive switching pulse was applied (+6 V) on a smaller inner 250 × 250 nm^2^ square of that region which turned into a large matrix of dark region (Fig. 3(e)). These results indicate the ferroelectric nature in BT-BCN film which is responsible for retention and fast switching in NVM devices. The ferroelectric nature further helps to realize re-writing in our systems because it is achieved by changing the polarization direction of ferroelectric materials (Supplementary Fig. S1 online).

### Interfacial characterization using HRTEM

**Figure 4:**
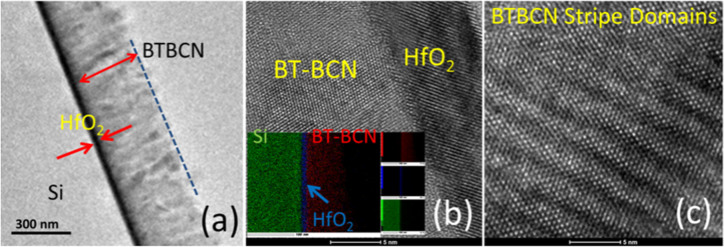
Interface study using high resolution transmission electron microscopy (HRTEM). (a) Cross sectional TEM image of BT-BCN on HfO_2_ buffered Si. Both the interfaces were found to be smooth and uniform; (b) High resolution TEM image of the BT-BCN and HfO_2_ interface. (inset) Elemental mapping in the BT-BCN/HfO_2_/Si interfaces using high resolution EDS-TEM. No interfacial defects as well as interdiffusion of Si can be observed at the interface or even in the active BT-BCN thin film. Therefore, ALD HfO_2_ suppressed the interdiffusion of Si into the BT-BCN, which may lead to good electrical performances from the devices; and (c) HRTEM image of BT-BCN stripe nano-domains, which is helpful for fast switching.

In NVM devices, factors like poor interface, diffusion of Si into the ferroelectric layer, and interfacial layer regrowth between the Si and high-k can generate defects leading to poor electrical performance of the device. Therefore, a good interface is critical for minimizing the charge injection from the interfacial layer formed due to reaction between the ferroelectric film and Si substrate. Figure 4(a) shows the cross-sectional high resolution transmission electron microscopy (HRTEM) micrograph of the BT-BCN/HfO_2_/Si stacks. The thickness of HfO_2_ and BT-BCN are estimated to be 10 nm and 300 nm, respectively, and these values were also confirmed by spectroscopic ellipsometer. Figure 4(b) shows the high resolution micrograph across the HfO_2_/BT-BCN interface to observe the process induced defects. This micrograph demonstrates the high crystalline quality of the stacks with atomically smooth, sharp interface and chemical stability between the HfO_2_ and the BT-BCN layers. The growth of HfO_2_ was found to be crystalline which was also seen from the XRD spectrum. However, the BT-BCN thin film had columnar growth with grains having different orientations. The absence of lattice fringes in nearby area can be understood by assuming change in diffraction conditions and deviation from the zone axis. No distinguishable interdiffusion or secondary phase formation was observed in the BT-BCN layer (inset of Fig. 4(b)). This indicates that insertion of ALD HfO_2_ between the ferroelectric layer and the Si prevents the diffusion of Si into the ferroelectric layer, and also suppresses the interfacial layer growth below BT-BCN (see Supplementary Fig. S2 and Tables S2 and S3 online). A contrary result has been obtained when BT-BCN was directly deposited onto Si (100) without any buffer layer (see Supplementary Fig. S3 online). From that micrograph, it can be observed that Si was interdiffused into BT-BCN and the interface was found to be very poor. The high resolution micrograph of stripes of ferroelectric nano-domains present in BT-BCN is shown in Fig. 4(c). Similar stripe nano-domains were observed in bulk BTO and PbTiO_3_ thin films^15^,^28^,^29^. The small size of nano-domains has been found to be associated with high mobility, which results in high domain wall density increasing the piezoelectric response of the system. We believe that these nano-domains are responsible for the fast switching^23^ as observed in the PFM studies.

### Band-alignments study

**Figure 5:**
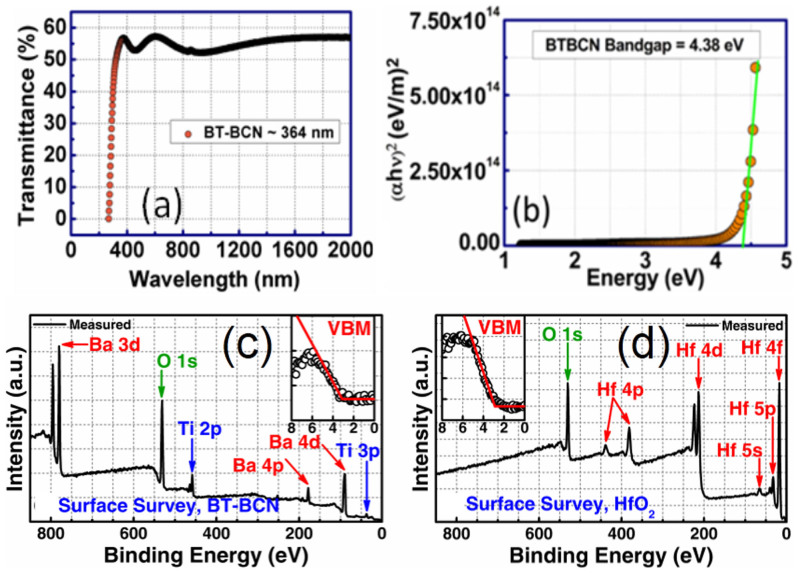
Optical band-gap studies of BT-BCN thin films and x-ray photoelectron spectroscopy (XPS) survey spectra of BT-BCN and HfO_2_. (a) Transmission spectra for BT-BCN thin films deposited on glass substrate. A sharp absorption peak found around 364 nm can be attributed to the direct band-to-band transition; (b) (*αhν*)^2^ vs *hν* to determine the optical bandgap of BT-BCN and it was found to be 4.38 eV; (c) Survey spectrum for BT-BCN thin film. (inset) VBM of BT-BCN thin film; and (d) survey spectrum for HfO_2_ thin film. (inset) VBM of HfO_2_ thin film. The concentration of Cu and Nb in the BT-BCN film was too low to detect via XPS.

**Figure 6:**
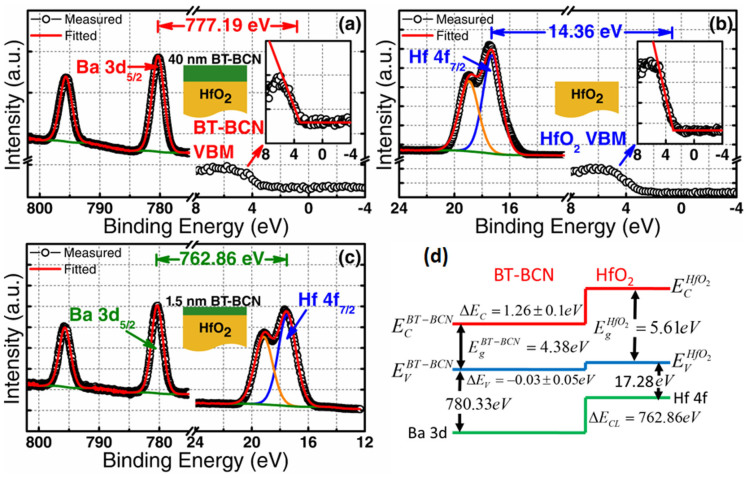
Determination of BT-BCN/HfO_2_ heterojunction band-alignments using x-ray photoelectron spectroscopy (XPS). XPS spectra of (a) Ba 3d CL and VBM of 40 nm BT-BCN thin films on HfO_2_; (b) Hf 4f CL spectrum and VBM of HfO_2_ film; (c) Ba 3d and Hf 4f CL spectra of 1.5 nm thin BT-BCN/HfO_2_ interface; and (d) Energy banddiagram of BT-BCN/HfO_2_heterointerface obtained from XPS measurements. The *ΔE_V_* was measured and the *ΔE_C_* was calculated based on the measured *ΔE_V_* and the difference in bandgap of HfO_2_ and BT-BCN. The *ΔE_C_* refers low leakage current in the devices.

Although BT-BCN has emerged as new functional oxide material, its bandgap data is not available in the literature. In order to understand the electrical properties of the Al/BT-BCN/HfO_2_/p-Si NVM devices, it is very important to determine the band alignment properties of these stacks. The band alignment of Si/HfO_2_ has been studied by prior researchers and the conduction band-offset (*ΔE_C_*) and valence band-offset (*ΔE_V_*) data between Si and HfO_2_ is available in literature^30^. However, the band alignment results between HfO_2_ and BT-BCN is not available. In this work, we have determined the band gap of the BT-BCN using a UV-vis-NIR spectrometer and the transmission spectrum of BT-BCN as shown in Fig. 5(a). A sharp absorption peak around 364 nm was found and it is attributed to the direct band-to-band transition. In order to calculate the direct bandgap, we used the Tauc's relationship *αhν* = *C*(*hν* − *E_g_*)*^n^*, where *α* is a absorption coefficient, h*ν* is the energy of the incident light, C is a constant, E_g_ is the bandgap, and n is the index (for direct transition, n = ½; for indirect transition, n = 2). Figure 5(b) shows the (*αhν*)^2^ vs *hν* plot where the linearity in the absorbance range confirms the direct transition and the bandgap was determined by extrapolation (*αhν*)^2^ = 0 resulting in a value of 4.38 eV. The band alignment properties of the BT-BCN/HfO_2_ heterostructure were determined using XPS by measuring three samples: (1) 40 nm BT-BCN on 25 nm HfO_2_, (2) 25 nm HfO_2_, and (3) 1.5 nm BT-BCN on 25 nm HfO_2_. The binding energy separation between an atomic core level (CL) and the valence band maximum (VBM) for each oxide was determined using XPS spectra of films whose thickness exceeded the photoelectron escape depth for photoelectrons generated in the underlying layer or at the interface, i.e. samples (1) and (2), respectively. Measurement of such thick samples resulted in CL and VBM spectra representative of only a singular oxide species, eliminating the influence of interfacial band-bending and underlying films on the acquired photoelectron signal. Band-bending at the BT-BCN/HfO_2_ interface was accounted for separately by collection of CL spectra from a thin (less than the photoelectron escape depth for photoelectrons generated in the HfO_2_) BT-BCN layer deposited on thick HfO_2_, i.e. sample (3). The band-bending-induced shifts in the atomic CL at the interface and the associated CL separation could then be used in the determination of *ΔE_V_* in conjunction with the measured thick-film binding energy separations. The XPS survey spectra were recorded from thick BT-BCN sample (1) (Fig. 5(c)) and thick HfO_2_ sample (2) (Fig. 5(d)) for a binding energy range of 0 eV to 850 eV. For clarity, the magnified VBM for BT-BCN and HfO_2_ have been included in the inset of Fig. 5(c) and Fig. 5(d), respectively. The lack of Cu- and Nb-related CL peaks in Fig. 5(c) suggests a concentration of Cu and Nb (x_total_ = 0.025, 
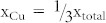
 and 
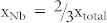
) that is too below the XPS detection limits. To verify the presence of Cu and Nb within the as-deposited BT-BCN, secondary ion mass spectroscopy (SIMS) depth profiling was performed and the presence of Cu and Nb was identified (see Supplementary Fig. S4 online). Figure 6(a) shows the thick Ba 3d CL spectrum and VBM of BT-BCN film, whereas, Fig. 6(b) shows the Hf 4f CL spectrum and VBM of HfO_2_ film. Figure 6(c) shows the Ba 3d CL and Hf 4f CL spectrum of 1.5 nm BT-BCN on HfO_2_. The *ΔE_V_* at the BT-BCN/HfO_2_ heterointerface was determined using these measured CL spectra with the following equation^31^,^32^,^33^,^34^
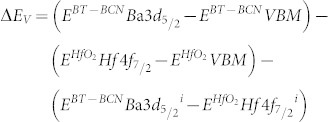
where 
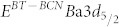
 and 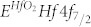 are the CL binding energies for the thick BT-BCN and HfO_2_ films, respectively, *E^BT−BCN^VBM* and 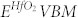 is the VBM for each material, and 
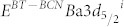
 and 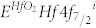 are the respective CL binding energies taken at the interface of BT-BCN and HfO_2_. The *ΔE_C_* at the BT-BCN/HfO_2_ interface was obtained from the following equation: 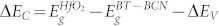, where 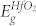 and 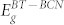 are the bandgap of HfO_2_ and BT-BCN, respectively. The CL binding energies were taken as the FWHM (full width-half maximum) of the associated peak (see characterization for more information). Spin-orbit coupling peak doublet areas were constricted during curve fitting. The curves were fitted using CasaXPS v2.3.14 using a Shirley-type (i.e. non-linear) background and a Lorentzian convolution fitting. The VBM was determined via the linear extrapolation method (as used in Tauc plots), where the intersection of the linear edge of the VBM and the background is the VBM binding energy. The choice of error bar in the VBM is a result of multiple regression fits and the variance in the selected position of the VBM binding energy resulting from one fit to the other^32^,^34^,^35^. The uncertainties are given in the calculations. The VBM values are determined by linear extrapolation of the leading edge to the base line of the VB spectra recorded on the BT-BCN and thick HfO_2_ film to the base line. The energy difference between the Ba 3d centroid and BT-BCN VBM was determined to be 777.19 ± 0.05 eV (as shown in Fig. 6(a)). The energy difference between Hf 4f centroid and the Hf VBM was found to be 14.36 ± 0.05 eV (as shown in Fig. 6(b)). The energy difference between Ba 3d centroid and the Hf 4f CL was found to be 762.86 ± 0.05 eV for the sample where 1.5 nm BT-BCN film was deposited on HfO_2_ film (as shown in Fig. 6(c)). Therefore, the *ΔE_V_* for HfO_2_/BT-BCN interface was determined to be −0.028 ± 0.05 eV. The bandgap of BT-BCN was found to be 4.38 eV, whereas the bandgap of HfO_2_ was taken from our recently published literature where it was reported to be 5.61 eV^32^,^34^. The *ΔE_C_* of BT-BCN/HfO_2_ interface was calculated to be 1.26 ± 0.1 eV. Figure 6(d) shows the schematic band alignment of the BT-BCN and HfO_2_ heterojunction based on the above results. The band alignment values between BT-BCN and HfO_2_ are tabulated in Table 1. The obtained *ΔE_C_* and *ΔE_V_* values suggest that the barrier height of BT-BCN and HfO_2_ interface was large enough to obtain low leakage current. In addition, these values also help to design a complete band diagram of BT-BCN/HfO_2_/Si NVM devices.

**Figure 7:**
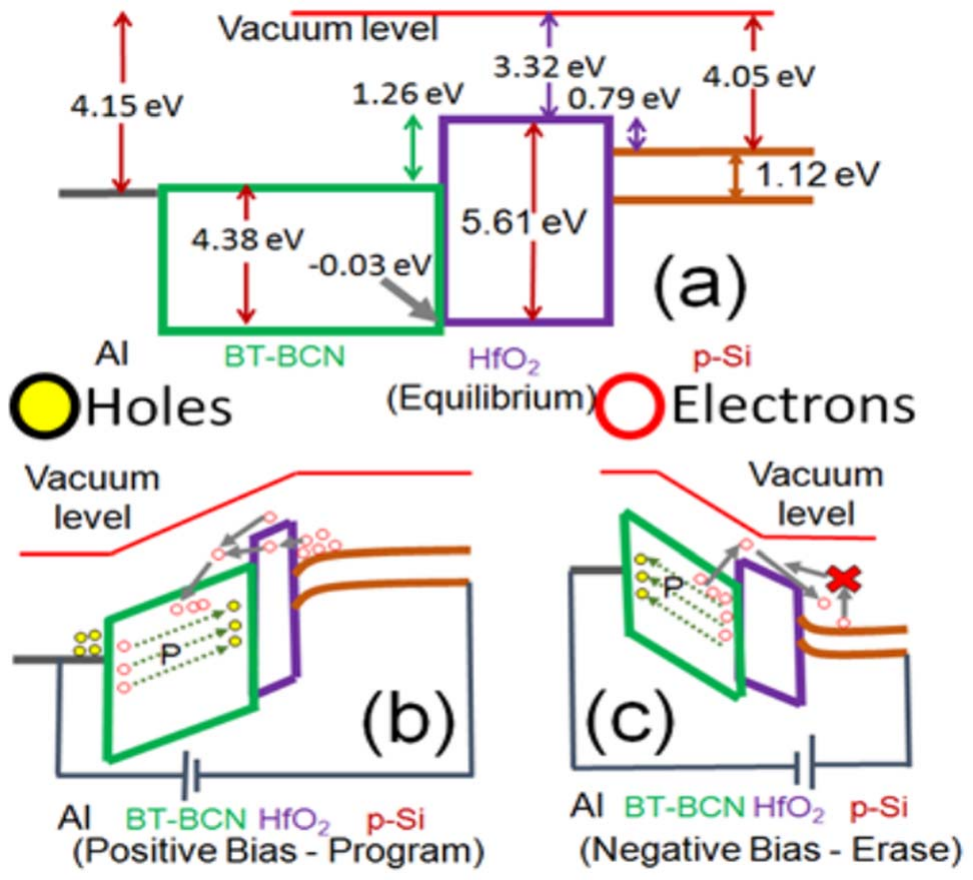
Energy banddiagram of Al/BT-BCN/HfO_2_/p-Si NVM devices. (a) in equilibrium, i.e., when no voltage is applied; (b) When positive voltage was applied at the metal gate with respect to the Si, considered as program operation; and (c) When negative voltage was applied at the metal gate with respect to the Si, considered as erase operation.

The band-diagram of these devices are proposed and utilized to develop the equilibrium program and erase operations as shown in Figs. 7(a), 7(b), and 7(c), respectively. For the proposed device structure, the program operation can be conducted by applying the positive voltage at the gate. When the gate bias is positive, the ferroelectric film is polarized and the Si surface is in inversion mode (as shown in Fig. 7(b)). The inverted electrons in the conduction band of silicon can be injected into the 10 nm HfO_2_ through Fowler-Nordheim tunneling^36^ and get trapped in BT-BCN. Thus, the obtained memory or retention property is due to these trapped carriers. In addition, both the remnant polarization and ferroelectric switching property of BT-BCN have strong influence on the programming operation of this device. In the presence of high positive voltage, a neutral oxygen vacancy inside the BT-BCN film can also release free electrons. These free electrons are driven by polarization switching or domain wall movement and an extra narrow electron region can be formed inside the ferroelectric^37^. These could also facilitate the program operation. The erase operation can be done by applying negative voltage at the gate (as shown in Fig. 7(c)) and it can be realized in two ways. First, when the gate bias is negative, the trapped electrons will be emitted back to Si. Moreover, applying negative bias at the gate allows ferroelectric polarization to switch direction and drive the extra electrons toward Si surface. At this time, the neutral oxygen vacancies also lose electrons and leave holes in the BT-BCN film. Under negative voltage, an extra number of positive charges will also be induced at the silicon surface due to accumulation mechanism. The Si conduction band bends upward and no electrons can travel from the Si surface to the ferroelectric layer due to the existence of potential barrier. Secondly, due to a large valence-band offset at the HfO_2_/Si interface for holes, few holes can jump across the barrier and get neutralized when they come across the trapped electrons^30^. These two reasons are responsible for erase operation and it can be accomplished as a very fast process, thus leading to low power dissipation through the device.

### Electrical characterization

**Figure 8:**
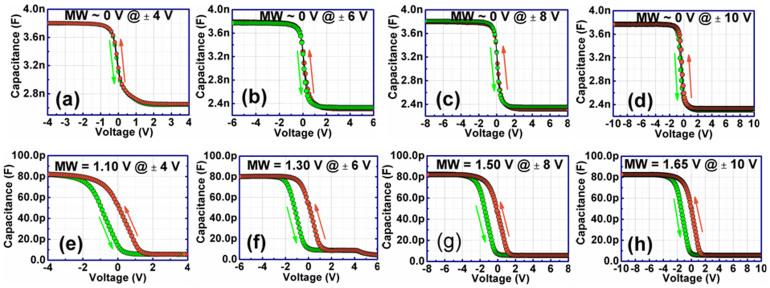
Capacitance-voltage (*C-V*) characteristics of Al/HfO_2_/p-Si devices at different voltages. (a) 4 V, (b) 6 V, (c) 8 V, and (d) 10 V. When BT-BCN is not present, no memory window (MW) obtained from these *C-V* characteristics. Capacitance-voltage (*C-V*) characteristics of Al/HfO_2_/p-Si devices at different voltages (e) 4 V, (f) 6 V, (g) 8 V, and (h) 10 V. MW increased from 1.10 V to 1.65 V when the applied dc voltage increased from 4 V to 6 V. Therefore, the memory window was originated due to the presence of BT-BCN ferroelectric film between metal gate and HfO_2_ buffer layer.

We fabricated Al/BT-BCN/HfO_2_/p-Si(100)/Al devices to study the electrical characteristics in order to understand the device performance. Figure 8 shows the high frequency (1 MHz) capacitance-voltage (*C–V*) characteristics of ferroelectric embedded NVM devices under various sweeping gate voltages. The gate voltage was swept from negative to positive voltage and then again back to negative voltage. The memory window, i.e., the flatband voltage (*V_FB_*) difference (between the *V_FB_* of the forward curve and that of the backward curve) measures the hysteresis voltage^38^. The *C-V* curves were not bent even after applying the negative voltage. Thus, good saturation characteristics of *C-V* curve indicate that both the leakage current and fixed oxide charges in the dielectric have been reduced. In a ferroelectric based NVM devices, the polarity of the program/erase gate voltage determines the polarization state of the ferroelectric layer, which in turn controls the accumulation or depletion of carriers in the semiconducting channel, giving rise to characteristic capacitance hysteresis. To understand the efficacy of BT-BCN ferroelectric thin film on memory window, we have prepared few control samples where BT-BCN was absent. The control sample is basically Al/HfO_2_/p-Si metal-oxide-semiconductor devices, i.e., there is no ferroelectric layer in between metal and HfO_2_. In control samples, the memory window was found to be close to 0 V, i.e., very poor memory window for all the applied voltages (as shown in Figs. 8(a) – 8(d)). However, when the BT-BCN layer was inserted between Al and HfO_2_, the memory window was greatly increased and it was found to be 1.10, 1.30, 1.50, and 1.65 V, for 4, 6, 8, and 10 V applied voltages, respectively as shown in Figs. 8(e), 8(f), 8(g), and 8(h). This behavior suggests that hysteresis characteristics is due to ferroelectric domain reversion in BT-BCN films, and also exhibits that a significant number of charge trapping sites exists inside the ferroelectric layer, mainly due to the presence of Ti^3+^, which is basically considered as deep trap center. Thus, under positive bias, electron injection from the substrate can be referred as programming-mode, while hole injection under negative voltage can be referred as erasing-mode of operation. One can find a relation between the memory window and the applied voltage as obtained from *C-V* characteristics. When the sweeping voltage increases from ±4 V to ±10 V, the memory window increased from 1.10 to 1.65 V due to higher saturation level of the ferroelectric polarization. The memory window keeps increasing up to ±10 V and then gets saturated. However, it does not start to decrease even when the voltage was applied beyond ±10 V [see Supplementary Fig. S5 online]. The contrary results were obtained in some ferroelectric based NVM devices reported earlier where the memory window decreased after a certain voltage^3^,^9^,^30^,^39^. The decrease of memory window was ascertained to be the charge injection and build of depolarization field at the ferroelectric/semiconductor interface. Thus, in our present study, the obtained results suggest that charge injection has no influence on our fabricated memory devices and it can be believed that formation of a good interface between ferroelectric and Si basically suppressed the generation of depolarization field. It is also interesting to note that the memory window or hysteresis has not been changed when the scanning speed of bias voltage was changed from 0.01 V/s to 0.75 V/s in the *C–V* measurement. Therefore, it confirms that the memory window in our devices was originated only from the ferroelectricity of BT-BCN film, not from the mobile ionic charges^4^. For metal-oxide-semiconductor (MOS) FET device operation, ~0.1 V voltage is required to change the drain current by a factor of 10 in the subthreshold region^4^. Thus, the current on/off ratio can be expected on the order of 10^10^ from the lowest MW width of 1.10 V found in our devices. Since, the lowest memory window was obtained at 4 V, hence, this applied voltage is good for FeRAM applications^4^.

**Figure 9:**
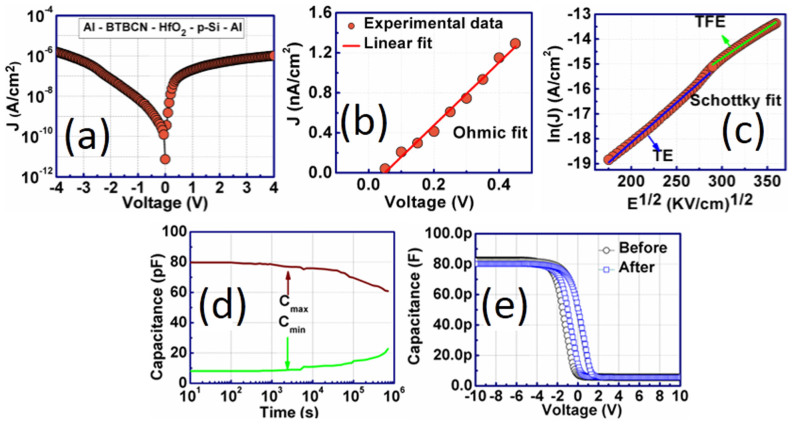
Leakage current and retention characteristics of Al/BT-BCN/HfO_2_/p-Si/Al NVM devices. (a) current density-voltage (*J-V*) characteristics, (b) Ohmic fitting for the current conduction, and (c) Schottky fitting for the current conduction. The leakage current was found to be ~10^−9^ A/cm^2^ at −1 V which is considered as very low leakage in ferroelectric memory. The leakage current was fitted to know which transport properties are responsible for leakage. From 0.1 V to 0.5 V, the current was due to Ohmic conduction, whereas 0.6 V to 4 V, both the thermionic and thermionic field emission were responsible for current transport; (d) Capacitance-time plot to know the charge retention property as a function of time. The device can retain up to 77% charge from its initial position even after 10^6^ s; and (e) *C-V* characteristic was measured again after the retention measurement and compared with the *C-V* characteristics which was measured earlier before the retention measurement. There was negligible shift of the *C-V* curve, shows its reliability.

The current density – voltage (*J-V*) characteristics of these memory devices is important in order to understand its leakage performance. The retention property can be greatly increased if the leakage is reduced. The *J-V* characteristics of Al/BT-BCN/HfO_2_/p-Si NVM devices is shown in Fig. 9(a). From the figure, the current density was found to be 7 × 10^−9^ A/cm^2^ at −1 V, which is considered as very low leakage when compared with other ferroelectric based memory devices available in literature [see Supplementary Table S1 online]. A very good interface between HfO_2_ and BT-BCN without any interdiffusion and smooth and dense structure of HfO_2_ and BT-BCN films with very low surface roughness have large impact on reducing the leakage current, as we can see in atomic force micrographs (see Supplementary Fig. S6 online). To gain more insight and to explain leakage current conduction mechanism, we have divided the *J-V* curves in two regions. In Fig. 9(b), we can see that the leakage current fits Ohmic characteristics, i.e., from 0 to 0.5 V, the obtained current transport is due to Ohmic conduction. On the other hand, beyond 0.5 V, the leakage current fits the Schottky emission (as shown in Fig. 9(c)). The current density – voltage relation for Schottky emission is given by 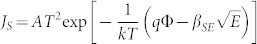, where *A* is a constant, *T* is the temperature, *k* is a Boltzmann's constant, *q* is the electronic charge, Φ is the potential barrier height, and *E* is the electric field. *β_SE_* is given by 
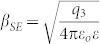
, where *ε_0_* is free space permittivity, and *ε* is the dielectric constant. From the Fig. 9(c), we can find out one interesting phenomenon, i.e., there are two slopes present in the Schottky fit. Thus, when the field was low, thermionic emission is mainly responsible for current transport which is marked with blue linear fit (lower part). However, when the field was increased more, the thermionic field emission has better impact on the transport property of these devices, which is shown in second linear fit in green color (upper part). In our fabricated devices, the thickness of BT-BCN was 300 nm. Hence, it is worthy to mention that the tunneling mechanism has no bearing on transport property in this case. To examine the memory property and stability of our fabricated memory devices, we have performed data retention characteristics of these devices as a function of time (as shown in Fig. 9(d)). The data retention for Al/BT-BCN/HfO_2_/p-Si NVM devices was measured at room temperature by biasing the devices to their respective flatband voltages. The devices were driven with the write voltage of ±6 V in height and 100 ms width followed by the time dependent measurements of high (*C_max_*) and low (*C_low_*) capacitances by keeping the bias fixed at the 0.5 V at which a maximum difference between the *C_max_* and *C_low_* capacitances was observed. We have focused on ±6 V because the same voltage was poled during PFM switching. The retention time can be determined when the difference between capacitances of the high and low states becomes half of its initial value with time^10^. It is interesting to observe that one can easily distinguish the high and low capacitance values even after ~10^6^ s. The *C_max_* was reduced from 79.80 pF to 60.85 pF, whereas the *C_low_* was increased from 8.11 pF to 22.62 pF. The long term retention is mainly due to the good ferroelectric and remnant polarization properties of BT-BCN. Other motivation factors, such as low depolarization field, lower interfacial reaction between Si and ferroelectrics, suppression of interfacial layer formation between Si and buffer layer, lower leakage current, smooth interfaces, and the dense structure of BT-BCN and HfO_2_ are also responsible for low leakage and high retention property. In order to investigate the reliability of our devices, *C-V* characteristics was performed after the retention measurement and it was found that the memory window saw an insignificant decrease from 1.65 V to 1.52 V (as shown in Fig. 9(e)). Therefore, aging time and voltage stress has negligible impact on the reliability of our fabricated devices.

## Discussion

We have successfully demonstrated the ferroelectric switching behavior of BT-BCN thin films and successfully integrated on HfO_2_ buffered Si for NVM device applications. PFM results suggest that polar domains in BT-BCN can be read and written with domains inversion by applied bias. HRTEM results reveal that there was no interdiffusion into the BT-BCN active material and smooth interfaces were achieved in our devices. Stripes of nano-domains were found in BT-BCN and the domain wall exhibited the 180° rotation of the polarization. The band alignment properties of BT-BCN on HfO_2_ were investigated using XPS. From these investigations, the obtained *ΔE_C_* and *ΔE_V_* offer an important guidance towards design of band diagram in order to understand the device operating principles. *C–V* measurements were incorporated to obtain the memory window and the maximum memory window was found to be 1.65 V when ±10 V was applied at the gate. If we keep increasing the gate voltage beyond ±10 V, the memory window does not change, i.e., charge injection or the depolarization field has negligible impact on our fabricated devices. On the other hand, the leakage current was found to be 7 × 10^−9^ A/cm^2^ at −1 V. It is noteworthy to mention that the three mechanisms, i.e., Ohmic conduction, thermionic emission and thermionic field emission are responsible for leakage current in our devices. The charge retention property was investigated and the devices offer good charge retention over time. The high and low capacitance values were clearly distinguishable even after ~10^6^ s. Thus, we successfully introduced a novel ferroelectric material BT-BCN and also demonstrated its potential application in NVM devices. Further optimization on the BT-BCN film thickness, different O_2_ partial pressure variation during growth and annealing, and insertion of a perovskite buffer layer (say SrTiO_3_ or La_0.7_Sr_0.3_MnO_3_) on Si could improve the fast ferroelectric switching and device performances. Though the flash memories are dominating current share markets, the present study introduces a novel ferroelectric thin film BT-BCN that can pave the way towards developing futuristic ferroelectric based NVM devices on Si platform for high-speed and low power electronic applications.

## Methods

### Materials preparation

The target (for PLD) of (1-x)BaTiO_3_-xBa(Cu_1/3_Nb_2/3_)O_3_ (x = 0.025) ferroelectric material was synthesized using the conventional solid state reaction method. For this, stoichiometric amounts of TiO_2_ (Alfa aesar, 99.0%), CuO (Alfaaesar, 99.0%), BaCO_3_ (Alfa aesar, 99.0%), Nb_2_O_3_ (Alfa aesar, 99.0%) were ball milled under ethanol for 24 h followed by drying at 80°C for 6 h. The powder thus obtained was calcined at1000°C for 2 h followed by ball milling for 48 h under ethanol. Subsequently, the powder was pressed in to a cylindrical target using uniaxial pressing. This target was further subjected to isostatic pressing (CIP) to achieve high green density. This cylindrical target was sintered at 1350°C for 2 h to achieve highly dense (>97%) body and the density of the target was measured by conventional Archimedes technique. The quality of materials was analyzed by XRD (see Supplementary Fig. S7 online).

### Device Fabrication

p-type Si (100) wafers were degreased by acetone and IPA, and finally rinsing in deionized (DI) water for 1 m. Then, the substrates were subjected to etch in a solution of HF-H_2_O in the ratio of 1:10 for 3 m to remove native oxides and terminate the surface dangling bonds with H atoms that causes surface passivation effect. The samples were finally dried by N_2_ gun.10 nm HfO_2_ films were grown by ALD in a Cambridge NanoTech system on Si (100) using a tetrakis(dimethylamino)hafnium compound as Hf precursor and H_2_O as oxygen source. During the HfO_2_ growth, the surface temperature of all Si samples and Hf precursor temperature were kept constant at 250°C and 75°C, respectively. The BT-BCN thin films were deposited by pulsed laser deposition (PLD) technique using a KrF excimer laser (λ = 248 nm) on HfO_2_/Si at a deposition rate of 0.5 Å/s using the synthesized BT-BCN target. To avoid contamination, we have minimized the queue time between ALD and PLD depositions. We loaded the sample into the PLD chamber for BT-BCN deposition immediately following the ALD HfO_2_ deposition. The focused laser beam irradiates the rotating target at 89 rpm with a laser energy density of ~2.5 J/cm^2^ at a repetition rate of 10 Hz. The deposition was made using a vacuum chamber with an oxygen pressure of 100 mTorr and the temperature was maintained at 750°C during the deposition of BT-BCN films. 100 nm thick aluminum (Al) gate electrodes and back side Ohmic contacts were formed on a substrate with a patterned shadow mask under a vacuum of 10^−6^ Torr using Kart Lesker PVD 250 electron beam evaporator. It was followed by rapid thermal annealing at 300°C for 3 min in FGA ambience. The device schematic is described in Supplementary Fig. S8 online.

### Characterizations

The surface morphology was studied using an atomic force microscopy (AFM) (Bruker, Dimension Icon, USA). To investigate the crystalline tetragonal structure of deposited BT-BCN, x-ray diffraction (XRD) pattern was recorded using a PANalytical X'Pert X-ray diffractometer (Cu Ka radiation) with pixel3D detector at an operating voltage of 45 kV and a current of 40 mA. The sample was mounted on a vertical sample stage controlled by a goniometer with a 2θ angular resolution of 0.0002° and scanned for 2 h, values ranging from 15 to 75 degree. In order to investigate the polarization switching behavior of domains in BT-BCN, a positive dc bias of 0 and ±6 V was applied on BT-BCN films using a scanning tip (SCM-PIT, Bruker). Cross sectional high resolution transmission electron microscopy (HRTEM) was used to characterize the domains formation in BT-BCN and also to study the interface between HfO_2_ and BT-BCN. The HRTEM imaging was performed using FEI Titan 80–300 transmission electron microscope. For this purpose, the electron transparent foils of thin film cross sections of BT-BCN/HfO_2_/Si were prepared by a standard polishing technique, i.e., mechanical grinding, dimpling, and Ar+ ion beam-milling. The bandgap of BT-BCN film was determined from transmission spectra obtained by HitachiU-4100 UV-vis-NIR spectrophotometer. To determine the bandgap, we have deposited 300 nm BT-BCN onto quartz substrate for obtaining transmittance spectrum. All electrical measurements were carried out in air ambient using Keithley 4200 semiconductor characterization system and Agilent 4284 A Precision LCR meter. The band-alignments between BT-BCN and HfO_2_ layers were investigated using a PHI Quantera SXM XPS system with a monochromated Al Kα (energy of 1486.7 eV) X-ray source. All binding energy spectra were collected with a pass energy of 26 eV and an exit angle of 45°, including the CL spectra as well as the angle-integrated photoelectron energy distribution curves for the valence band maxima (VBM). All binding energy spectra collected were adjusted to the adventitious carbon peak – C1s – CL at 285.0 eV. Dynamic SIMS was performed using Cameca IMS-7f GEO with Cs^+^ as primary ion beam to determine the compositional profile of Ba, Ti, O, Cu, and Nb atoms in the BT-BCN thin films.

## Author Contributions

S.K. conceived the idea and wrote the paper. S.K. and S.P. designed the experiments. S.K., D.M., M.C., Y.Z. and N.N.H. carried out the experiments. P.B. supervised the retention and device reliability measurements. S.P. and M.K.H. supervised the whole research. All authors discussed the results and commented on the manuscript.

## Supplementary Material

Supplementary Information Supplementary info

## Figures and Tables

**Table 1:** CL to VBM binding-energy difference for BT-BCN and HfO_2_ grown on Si

		Measured band offsets of BT-BCN and HfO_2_	
Sample	Binding energy difference	*ΔE_C_*	*ΔE_V_*
40 nm BT-BCN	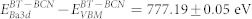		
HfO_2_	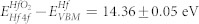		
1.5 nm BT-BCN on HfO_2_	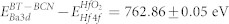		
		1.26 ± 0.1 eV	−0.028 ± 0.05 eV
