# Supplementary material for: Integration of lead-free ferroelectric on HfO2/Si (100) for high performance non-volatile memory applications
Source: Sci Rep. 2015 Feb 16;5:8494. doi: 10.1038/srep08494 (PMC4329549; doi:10.1038/srep08494)
Supplement: Supplementary Information — Supplementary info [file srep08494-s1.pdf]

Supplementary Information

Integration of lead-free ferroelectric on HfO_2/Si (100) for high performance non-volatile memory applications

Souvik Kundu[1], Deepam Maurya[1], Michael Clavel[2], Yuan Zhou[1], Nripendra N. Halder[3], Mantu K. Hudait[2], Pallab Banerji[4], and Shashank Priya[1]

[1]*Center for Energy Harvesting Materials and Systems (CEHMS), Department of Mechanical Engineering, Virginia Tech, Blacksburg, Virginia 24061, USA*

[2]Advanced *Devices & Sustainable Energy Laboratory (ADSEL), Bradley Department of Electrical and Computer Engineering, Virginia Tech, Blacksburg, Virginia 24061, USA*

[3]*Advanced Technology Development Centre, Indian Institute of Technology Kharagpur, Kharagpur 721302, India*

[4]*Materials Science Centre, Indian Institute of Technology Kharagpur, Kharagpur 721302, India*

Correspondence and requests for materials should be addressed to S.P. (email: spriya@vt.edu) or S.K (email: souvikk@vt.edu).

Prior researchers have attempted to implement ferroelectric materials onto Si[S1-S9]. However, these prior studies have not shown domain switching in their ferroelectric films. From Table S1, one can find that the highest retention time of 4×10^5 s was achieved using (Bi, La)$_4$Ti$_3$O$_{12}$ (BLT)/HfO_2, but it offers poor memory window of 0.9 V[S9]. On the other hand, $SrBi_2Ta_2O_9$ (SBT)/HfO_2 or SBT/ZrO_2 exhibited the memory window in the range of 0.87 V to 2 V and also shows poor retention performance of only 10^3 s[S1,S2]. However, SBT/HfO_2 shows a great promise for lowering leakage current compared with other counterparts[S5]. Very recently, $BiFeO_3$ (BFO) was introduced as an alternative due to its high remnant polarization. However, there is no report available in the literature related to the retention property using this material[S3]. Zhang et al. investigated the SBT/HfO_2 based MFOS memory devices and studied retention loss mechanism from SBT domains[S10]. However, the study was not extended to reveal the ferroelectric domain formation and their switching.

Table S1 | Summary of studied ferroelectric/oxide/Si memory devices

Ferroelectric/oxide	Thickness (nm)	MW (V)	Leakage Current @ 1V (A/cm^2)	Retention time (s)	Ref.
SBT/HfO_2	280/12	1.6	1×10^{-8}	5.4×10^{3}	S1
SBT/ZrO_2	160/45	2	8×10^{-7}	5.4×10^{3}	S2
BFO/TiO_2	250/150	1.11	6×10^{-9}	–	S3
SBT/HfTaO	300/4	0.65	1×10^{-7}	8.64×10^{4}	S4
SBT/HfO_2	300/6	0.87	1×10^{-12}	2.88×10^{4}	S5
SBT/STO	300/23	1.1	1.5×10^{-8}	8.64×10^{4}	S6
SBT/PrOx	400/32	0.3	1×10^{-9}	1×10^{4}	S7
PZT/Y_2O_3	290/11.8	1.5	3×10^{-5}	1×10^{4}	S8
BLT/HfO_2	380/15	0.9	2×10^{-9}	4×10^{5}	S9

To fabricate a high performance ferroelectric based memory devices, once should select a ferroelectric material which has (1) high remnant polarization so that it will offer reliable reading operation, (2) low coercive field and thus low programming voltage to operate, and (3) good switching capability and thus low energy loss. Among different ferroelectric materials, SBT and Pb(Zr,Ti)O_3 (PZT) are the most promising materials for NVM applications[S11]. The PZT material has very high coercive field allowing operations at higher programming voltage[S11]. SBT material has much lower remnant polarization as compared with PZT, allowing very low retention

property[S11]. It is also noteworthy that PZT material contains lead which is considered as toxic. Thus, searching for a lead free ferroelectric material consisting of higher remnant polarization, low coercive voltage, and excellent switching characteristics is desirable to facilitate the high performance NVM devices.

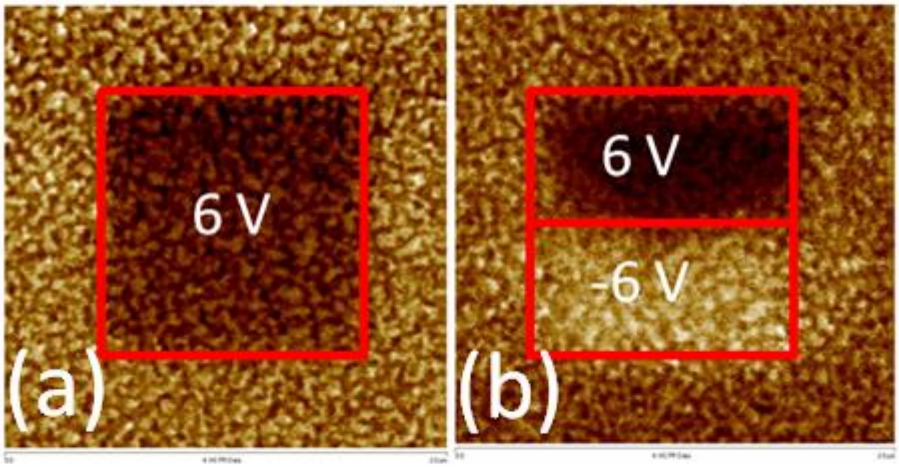

Figure S1 | Piezoelectric force microscopy images of BT-BCN films (a) poled with +6 V over 1μm region, and (b) poled with ± 6 V over 1 μm region.

Figure S1(a) shows the polarization switching of BT-BCN films after poling with +6 V, whereas, Fig. S1(b) shows the polarization inversion of BT-BCN films when poled with ± 6 V.

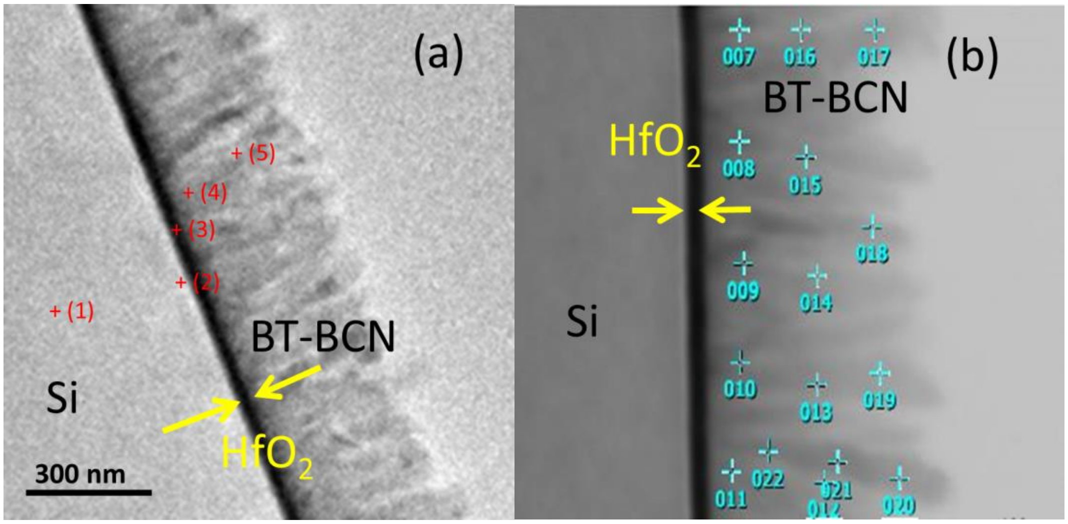

Figure S2 | EDS spot analysis in (a) Si/HfO_2/BT-BCN stack and (b) BT-BCN film to confirm the chemical uniformity.

Figure S2(a) shows the EDS spot analysis at different places in Si/HfO_2/BT-BCN stack and the quantified data are presented in Table S2. The spots (1), (2), (3), (4), and (5) are represented as Si, Si/HfO_2 interface, HfO_2, BT-BCN/HfO_2 interface, and BT-BCN, respectively. From this analysis, it was found that there is no significant cross-interdiffusion between Si and BT-BCN layer. Figure S2(b) shows the EDS spot analysis on BT-BCN film to confirm its large range chemical uniformity and the quantified data are presented in Table S3. This is also visible from the corresponding TEM image. Previous elemental results were confined in a local area as depicted in the corresponding TEM image (Fig. S2(a)).

Table S2 | Summary of elemental analysis at different spots in Si/HfO_2/BT-BCN stack

Spot	Element (atomic %)				
	O	Si	Ti	Ba	Hf
1	1.87	98.04	0.09	0.00	0.00
2	49.61	36.70	0.16	0.30	13.23
3	68.82	8.62	0.59	0.16	21.81
4	64.44	7.37	11.85	16.33	0.00
5	69.29	6.32	9.96	14.28	0.15

Table S3 | Summary of elemental analysis at different spots in BT-BCN thin film

Spot	Element (atomic %)				
	O	Si	Ti	Ba	Hf
7	66.90	5.77	11.98	15.34	0.00
8	66.18	5.51	12.90	15.41	0.00
9	67.68	5.12	12.31	14.89	0.00

10	66.21	5.68	11.18	16.93	0.00
11	65.61	5.59	12.75	16.05	0.00
12	69.73	5.47	11.33	13.47	0.00
13	70.32	5.45	9.75	14.48	0.00
14	69.26	5.25	11.76	13.73	0.00
15	68.55	5.76	12.11	13.58	0.00
16	66.63	5.48	11.70	16.19	0.00
17	64.52	6.57	11.75	17.16	0.00
18	68.28	7.19	11.71	12.82	0.00
19	65.61	5.55	11.42	17.42	0.00
20	64.03	4.25	13.76	17.96	0.00
21	67.22	6.08	11.11	15.59	0.00
22	68.93	4.12	10.29	16.66	0.00

The data obtained from Fig. S3 indicates that BT-BCN was directly deposited onto Si. This deposition was conducted in order to understand the effectiveness of HfO_2 buffered Si. From the Fig. S3, one can find that Si out diffusion occurred during the deposition of BT-BCN. The interface between BT-BCN and Si was found to be very rough which is not suitable for fabricating electronic devices. Thus, HfO_2 buffer layer plays a important role in providing high quality BT-BCN film suitable for practical devices.

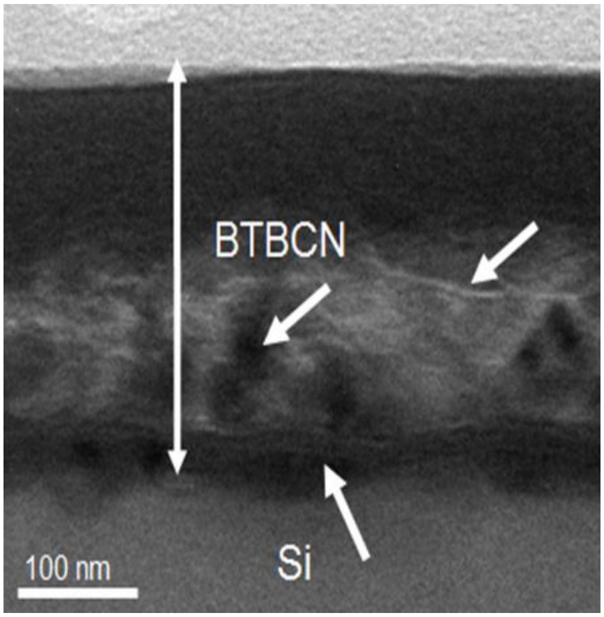

Figure S3 | Transmission electron microscope (TEM) micrograph of BT-BCN/Si interface. No buffer layer was used prior to the deposition of BT-BCN. Cross-interdiffusion occurred when BT-BCN was directly deposited on Si.

Secondary ion mass spectroscopy (SIMS) has been used to detect Cu and Nb in BT-BCN film. Figure S4 shows the detection of Cu and Nb during SIMS matrix profiling reinforces the dopant-induced $BaTiO_3$ phase change observed via XRD shown in Fig. 1(b) in the main manuscript,

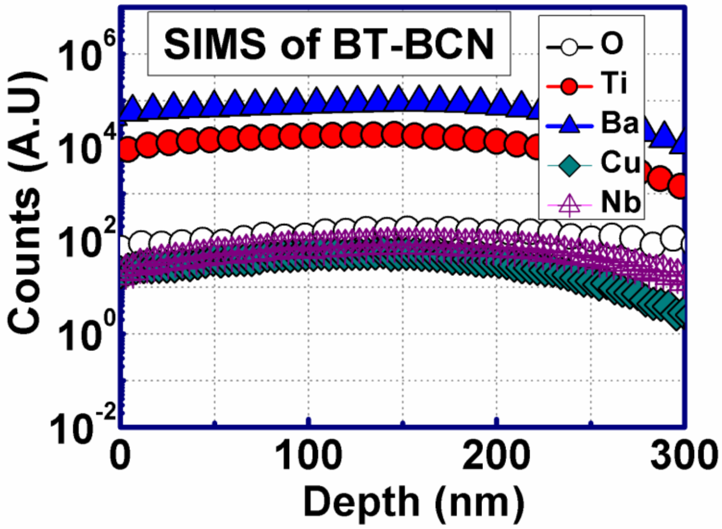

Figure S4 | SIMS depth profile of a BT-BCN film.

suggesting the successful incorporation and activation of Cu and Nb into the $BaTiO_3$ film.

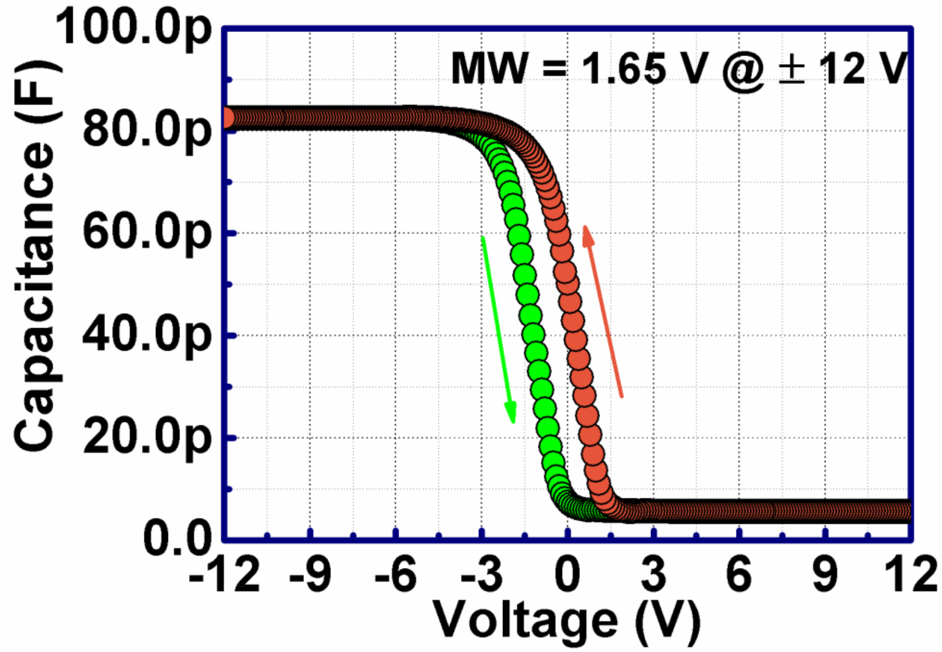

Figure S5 | Capacitance-voltage characteristics of Al/BT-BCN/HfO_2/p-Si with sweeping ±12 V. Memory window does not shift even if the higher voltage (> ±10 V) is applied.

Figure S5 shows the capacitance-voltage characteristics for Al/BT-BCN/HfO_2/p-Si devices. The memory window keeps increasing up to ± 10 V and then saturates. However, it does not start to decrease even when the voltage was applied beyond ± 10 V (here ± 12 V is shown).

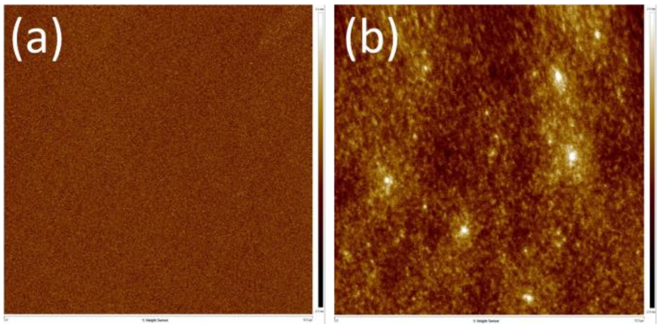

Figure S6 | Atomic force micrograph of (a) HfO_2 surface and (b) BT-BCN surface deposited on HfO_2/Si.

To analyze the surface morphology of HfO_2 and BT-BCN films, we have performed AFM analysis of such films as shown in Figs. S6(a) and S5(b), respectively. From the figures it can be

seen that both the HfO_2 and BTBCN films adhered well on the substrate and their surfaces were very smooth. The RMS roughness for HfO_2 was found to be 0.28 nm, whereas for BT-BCN it was 0.46 nm, which indicates a dense surface morphology.

Traditional x-ray diffraction (XRD) patterns (Fig. S7) were recorded for BT-BCN target to confirm the phase evolution using PANalytical X'Pert Pro powder X-ray diffractometer with CuKα radiation (λ =1.5418 Å). The scans were conducted in the range of 20 - 60° (2θ) operating at 45 kV and current of 40 mA.

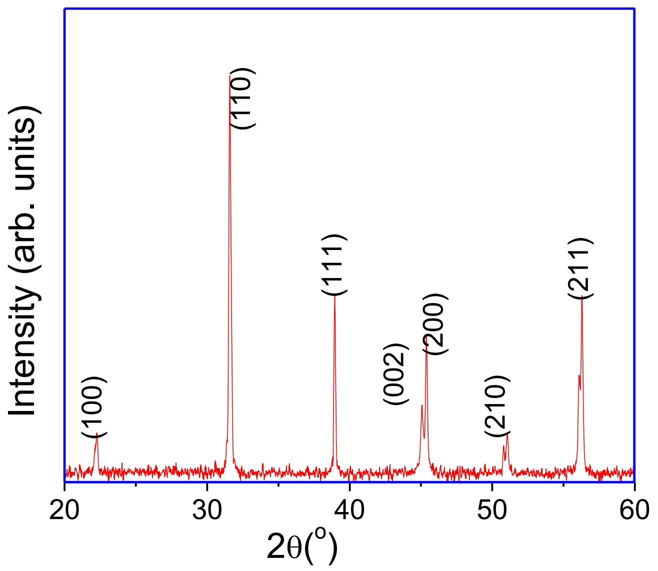

Figure S7 | XRD spectra of BT-BCN target.

The schematic of BT-BCN ferroelectric based NVM device structure is shown in Fig. S8. First a 10 nm HfO_2 was deposited onto Si using atomic layer deposition (ALD) technique, then pulsed laser deposition (PLD) was employed to deposit a 300 nm BT-BCN film on HfO_2/Si. Aluminum (Al) was used as top and bottom electrodes and was deposited by electron beam evaporation technique.

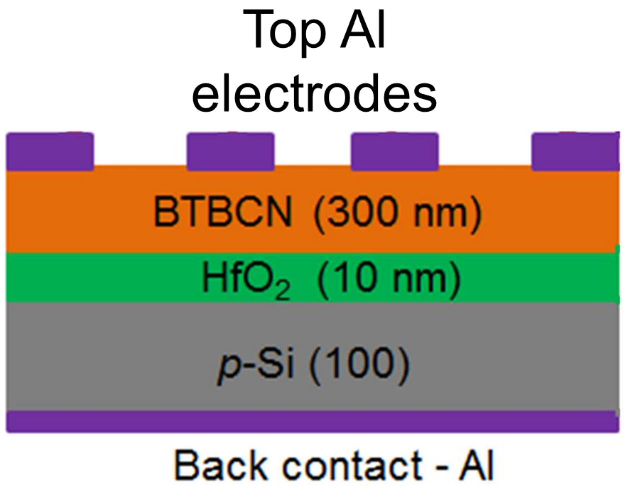

Figure S8 | Device schematic of Al/BT-BCN/HfO_2/p-Si NVM devices.

References

S1. Roy, A. et. al. Structural and electrical properties of metal–ferroelectric–insulator–semiconductor structure of Al/$SrBi_2Ta_2O_9$/HfO_2/Si using HfO_2 as buffer layer. *J. Phys. D: Appl. Phys.* **41,** 095408 (2008).

S2. Roy, A. et. al. Interfacial and electrical properties of $SrBi_2Ta_2O_9$/ZrO_2/Si heterostructures for ferroelectric memory devices. *J. Appl. Phys.* **104,** 064103 (2008).

S3. Xie, D. et. al. Characteristics of Pt/$BiFeO_3$/TiO_2/Si capacitors with TiO_2 layer formed by liquid-delivery metal organic chemical vapor deposition. *Appl. Phys. Lett.* **97,** 172901 (2010).

S4. Lu, X. -B. et. al. Characterization of HfTaO films for gate oxide and metal-ferroelectric-insulator-silicon device applications. *J. Appl. Phys.* **103,** 044105 (2008).

S5. Tang, M. H. et. al. Capacitance-voltage and retention characteristics of Pt/$SrBi_2Ta_2O_9$/HfO_2/Si structures with various buffer layer thickness. *Appl. Phys. Lett.* **94,** 212907 (2009).

S6. Lu, X. et. al. Characteristics of metal-ferroelectric-insulator-semiconductor diodes composed of Pt electrodes and epitaxial $Sr_{0.8}Bi_{2.2}Ta_2O_9$ (001)/$SrTiO_3$(100)/Si(100) structures. *J. Appl. Phys.* **105,** 024111 (2009).

S7. Noda, M. et. al. Basic characteristics of metal-ferroelectric-insulator-semiconductor structure using a high-k PrOx insulator layer. *J. Appl. Phys.* **93,** 4137 (2003).

S8. Shih, W. –C. Fabrication and characterization of metal-ferroelectric ($PbZr_{0.53}Ti_{0.47}O_3$)-Insulator (Y_2O_3)-semiconductor field effect transistors for nonvolatile memory applications. *J. Appl. Phys.* **103,** 094110 (2008).

S9. Park, B. –E. et. al. Five-day-long ferroelectric memory effect in Pt/$(Bi,La)_4Ti_3O_{12}$/HfO_2/Si structures. *Appl. Phys. Lett.* **85,** 4448 (2004).

S10. Zhang, Z. H. et. al. Retention loss in the ferroelectric ($SrBi_2Ta_2O_9$)-insulator (HfO_2)-silicon structure studied by piezoresponse force microscopy. *EPL.* **98,** 27011 (2012).

S11. Ferroelectric Dielectrics Integrated on Silicon [Defay, E. (ed.)] (Wiley, New Jersey, 2011).
